# The Expression Levels of Heat Shock Protein 90 (HSP90) in *Galleria mellonella* Following Infection with the Entomopathogenic Nematode *Steinernema carpocapsae* and Its Symbiotic Bacteria *Xenorhabdus nematophila*

**DOI:** 10.3390/insects16020201

**Published:** 2025-02-12

**Authors:** Davide Banfi, Maristella Mastore, Tommaso Bianchi, Maurizio Francesco Brivio

**Affiliations:** Laboratory of Applied Entomology and Parasitology, Department of Theoretical and Applied Sciences (DiSTA), University of Insubria, 21100 Varese, Italy; davide.banfi@uninsubria.it (D.B.); maristella.mastore@uninsubria.it (M.M.); tbianchi1@studenti.uninsubria.it (T.B.)

**Keywords:** heat shock proteins, HSP90, entomopathogen nematodes, *Steinernema carpocapsae*, *Xenorhabdus nematophila*, *Galleria mellonella*, climate change

## Abstract

Heat shock proteins (HSPs), particularly HSP90, play a vital role in insect responses to environmental and biotic stresses by maintaining protein stability and supporting immune defenses. This study explores HSP90 regulation in *Galleria mellonella* larvae following exposure to the nematode *Steinernema carpocapsae* and its symbiotic bacterium *Xenorhabdus nematophila*. Exposure to live nematodes caused slight changes in HSP90 expression, while non-viable nematodes had no effect, suggesting that nematode secretions or symbiotic bacteria do not directly influence HSP90 levels. However, nematodes with altered surface properties significantly increased HSP90 expression. *X. nematophila* also moderately elevated HSP90 levels but this effect disappeared when weakly bound surface proteins were removed. Interestingly, under thermal stress, live nematodes reduced heat-induced HSP90 expression, whereas surface-treated nematodes enhanced it. These findings suggest that HSP90 modulation is influenced by biological control agents, highlighting a potential link between HSP90 and immune detection of invaders. This interaction may be crucial in adapting biological control strategies in response to climate change. Further research is needed to clarify HSP activation pathways, host immune interactions, and mechanisms of entomopathogen immune evasion, particularly under varying environmental temperatures, to enhance bioinsecticide efficacy.

## Simple Summary

This study investigates how heat shock protein HSP90 helps *Galleria mellonella* larvae respond to stress from the nematode *Steinernema carpocapsae* and its symbiotic bacterium *Xenorhabdus nematophila*. Live nematodes slightly increased HSP90 expression, while dead nematodes had no effect, suggesting nematode secretions or bacteria do not directly impact HSP90. However, nematodes with altered surface properties significantly boosted HSP90 expression. *X. nematophila* also raised HSP90 levels, but this effect disappeared when weakly bound surface proteins were removed. Under heat stress, live nematodes reduced heat-induced HSP90 expression, whereas surface-treated nematodes increased it. These findings suggest that HSP90 plays a role in the immune system’s ability to detect and respond to biological threats. This interaction could be key for improving biological pest control methods, especially as climate change affects environmental conditions. Further research is needed to understand the pathways regulating HSP90 and how entomopathogens evade immune defenses, particularly under changing temperatures, to enhance the effectiveness of bioinsecticides.

## 1. Introduction

Heat shock proteins (HSPs) have been extensively studied across various fields, including transcriptional regulation, evolutionary biology, and stress response, since their initial discovery in *Drosophila melanogaster* due to an error in storage temperature [1]. These highly conserved proteins are essential for organisms in managing environmental stress, particularly changes in temperature. Research also shows that HSPs are upregulated in the presence of foreign organisms, protecting against biotic stressors [2,3,4]. In insects, HSPs primarily function as molecular chaperones, ensuring proper protein folding, preventing aggregation, assisting in the refolding or degradation of damaged proteins, and playing a crucial role in survival and adaptation to different habitats and climate change [5]. Physiological processes such as inflammation, immune response, and cellular damage lead to the upregulation of HSPs, helping host cells resist stressful events [2].

HSPs are categorized into seven families based on their molecular weight, including HSP70, HSP90, and small HSPs, each with distinct functions and regulatory mechanisms. Insects have four main HSP families, namely, sHSPs (small heat shock proteins), HSP60, HSP70, and HSP90; among them, HSP70 and HSP90 are the most studied [6].

The literature suggests that host HSPs also play a key role in defending against pathogen invasion [7,8]. Among HSPs, HSP90 is particularly critical for insect survival, responding to environmental challenges, maintaining cellular homeostasis, and supporting normal development; HSP90 modulates immune responses in the insect *Galleria mellonella*, contributing to larval resistance to pathogens [9,10,11]. High levels of HSP90 were observed in *Spodoptera frugiperda* cells under stress while a heat shock counteracted viral infection in *D. melanogaster* [12,13]. Biotic stresses, such as viral or fungal presence, activate small HSP genes via the eicosanoid pathway, and HSP synthesis is stimulated by pathogen-associated molecular patterns (PAMPs) in Coleoptera [14]. For instance, in the study conducted by Li et al. [15], both HSP90 mRNA and protein levels were overexpressed in the brain tissue of *Bombyx mori* following infection with *Bombyx mori* nucleopolyhedrovirus (BmNPV). There is also evidence that insect immune and stress responses may share signal transduction pathways [14,16,17]. However, although there are data on the effects induced by unicellular parasites, research on the expression of HSPs in insects when in the presence of pluricellular parasites such as nematodes is still very scarce. For instance, infection of adult *D. melanogaster* with axenic *Heterorhabditis bacteriophora* alters HSP gene expression, suggesting that nematodes, even if lacking their symbiotic bacteria, can modify host biological processes [18].

The lepidopteran *G. mellonella* Linnaeus 1758 (Lepidoptera: Pyralidae), also known as the greater wax moth or honeycomb moth, is a hive-destroying parasite of honeybees [19]. This organism is used as a model organism in a wide range of research fields, including toxicology, pharmacology, preclinical antimicrobial drug development, studies of virulence and pathogenesis, as well as in research on host–parasite interactions of biological control processes [19,20].

Among the organisms used in biological control are entomopathogenic nematodes and bacteria. These infectious agents typically act by profoundly altering the host’s physiology, causing its death [21,22]. In general, entomopathogens possess the ability to interfere with host immune processes by employing strategies that facilitate immune evasion. These strategies include immunoevasion mechanisms, such as molecular mimicry or molecular disguise, which prevent recognition by the host immune system at the discriminatory stage. Additionally, they can induce immunosuppression through the release of excreted or secreted factors that specifically target humoral and/or cellular components of the host’s immune system, thereby impairing its functionality [23,24]. Regarding entomopathogenic nematodes, lethal effects are observed during the later stages of infection, while the risk of being recognized and neutralized occurs early after infestation, involving surface components of the pathogen itself. As assessed in previous work [25], the body surface of *Steinernema feltiae*, particularly its epicuticular lipid moiety, plays a key role in immune evasion and immunodepression by removing certain *G. mellonella* hemolymph factors (host-interacting proteins, or HiPs) responsible for both non-self-recognition and the triggering of immune pathways, such as the proPO system, antibacterial peptides genes, encapsulation, and phagocytosis [25].

The present study aims to evaluate whether the nematode *Steinernema carpocapsae* (Nematoda, Rhabditidae) and its symbiont bacterium *Xenorhabdus nematophila* play a role in the up- or downregulation of HSP90 in *G. mellonella* larvae in the presence or absence of abiotic stresses (temperature), which are known to modulate stress protein expression [2]. HSP90 levels were assessed following injection of different bacterial species (*Micrococcus luteus, Escherichia coli,* live and salt-treated *X. nematophila*) and the live, cold-killed, surface-treated nematode *S. carpocapsae*. The time points at which HSP expression was evaluated corresponded to the interval during which the nematode exhibited its immunoevasive and immunosuppressive activities [26].

Modulation of HSP synthesis in insects offers critical insights into stress physiology and ecological adaptability, with substantial implications for their susceptibility to pathogens. Identifying the mechanisms that regulate the expression and function of HSPs could enhance the development of strategies to increase the effectiveness of biocontrol agents, including nematodes and entomopathogenic bacteria. 

## 2. Materials and Methods

### 2.1. Chemicals and Instruments

Microbial strains were supplied by the American Type Culture Collection, ATCC (Manassas, VA, USA). All reagents and media were supplied by Merck KGaA (Darmstadt, Germany), Cell Signaling (Danvers, MA, USA), and Thermo Fisher (Waltham, MA, USA). All materials, buffers, and solutions were autoclaved or filtered by 0.22 μm Sartorius Minisart filters (Goettingen, Germany). Instruments were supplied by Celbio Spa (Milan, Italy), Snijders Labs (Tilburg, The Netherlands), and Bio-Rad (Hercules, CA, USA). Centrifugations were carried out with a SIGMA 1–14 microcentrifuge (Osterode am Harz, Germany) and an Eppendorf 5804R (Hamburg, Germany) centrifuge. Optical density was determined by a Jasco V730 UV/Vis Spectrophotometer (Tokyo, Japan). Western blots were analyzed using a Uvitec Alliance Q9 and its relative software (Cambridge, UK).

### 2.2. Rearing Conditions and Maintenance of Galleria mellonella Larvae

Insect larvae were originally supplied by Kreka Ento-Feed (Ermelo, The Netherlands). Insect larvae were reared on a sterile artificial diet (9.5% rice flour, 9.5% oatmeal, 5% wheat germ, 14.8% torula yeast, 3.9% beeswax, 24% honey, 22.6% glycerol, 10.7% tap water, with no antimicrobial compounds or hormones added) and maintained in an insect climatic chamber at 26 °C with 60% RH (relative humidity) in the dark, according to the method of de Jong and colleagues [27]. For all assays, only healthy late-stage larvae, light yellow and weighing about 300–350 mg, were selected. Before use, larvae were sterilized with 70% ethanol.

### 2.3. Bacterial and Nematode Isolation and Host Infection

*G. mellonella* HSP levels were evaluated in the presence of various bacterial strains, as well as live, cold-killed, and surface-treated nematodes. 

*Micrococcus luteus* (ATCC 4698), *Escherichia coli* (ATCC 25922), and the symbiont *Xenorhabdus nematophila* were used to investigate the effects of bacterial infections on the host HSP90 levels. *E. coli* and *M. luteus* were used from a batch of lyophilized bacteria, while *X. nematophila* was isolated from its symbiont *S. carpocapsae* using a modified version of the experimental protocol described by Park and Kim [28]. 

Nematodes were obtained from a commercial product (Koppert Italia S.R.L., Bussolengo, Italy) using a sucrose density gradient separation followed by centrifugation for 10 min at 700× *g*. Collected nematodes were washed with 0.5% sodium hypochlorite and repeatedly rinsed with sterile tap water. To isolate *X. nematophila*, nematodes were resuspended in a Nematode Extraction Buffer (NEB; 100 mM Tris-HCl, 250 mM EDTA, 200 mM PMSF) and sonicated. The resulting suspension was streaked onto NBTA agar plates (37 g of nutrient agar, 25 mg of bromothymol blue, 1000 mL of distilled water, 4 mL of 1% 2,3,5-triphenyltetrazolium chloride), a nutrient-rich medium containing selective dyes that promote the growth of *X. nematophila* while inhibiting the growth of other microorganisms. Plates were incubated at 28 °C for 48 h and the characteristic bacterial colonies of *X. nematophila* were further subcultured on Luria-Bertani agar plates.

All the bacterial cultures were grown overnight at 37 °C in LB broth. The bacterial concentration was estimated by spectrophotometric measurement of absorbance at λ = 600 nm. Then cultures were centrifuged at 1000× *g* for 15 min, and the bacterial pellet was washed three times with sterile PBS (138 mM NaCl, 2.7 mM KCl, 10 mM Na_2_HPO_4_/KH_2_PO_4_, pH 7.4). Surface-treated *X. nematophila* was obtained by exposing the bacterial pellets to a high ionic strength buffer (500 mM NaCl in 10 mM Tris–HCl, pH 7.2) for 20 min, at room temperature. All bacterial strains were injected into *G. mellonella* larvae at a concentration of 10^3^ CFU/larvae and larvae were incubated at 26 °C (standard conditions).

Aliquots of isolated *S. carpocapsae* were cold-killed through sequential freeze–thaw cycles and mortality were monitored by a stereomicroscope. Aliquots of live *S. carpocapsae* were surface-treated with methanol–chloroform (1:2) to remove epicuticular lipids [29]. Nematodes were appropriately diluted and then injected into *G. mellonella* larvae (10 nematodes/larvae). All experimental groups were subjected to different incubation periods (30-, 60-, 90- and 120-min post-infection) at 26 °C (standard conditions) or 40 °C (thermal stress). 

The timing for assessing the expression of HSPs was chosen in accordance with data in the literature [16]. HSPs are expressed after abiotic or biotic stress, and their levels generally remain elevated throughout the duration of the stress before returning to baseline physiological levels. In our study, we evaluated the expression of shock proteins at 30, 60, 90, and 120 min after infection, both under standard conditions (26 °C) and after heat stress (40 °C). The timing of HSP activation tests also correlates with the period of immunological interaction that the nematode establishes with the host insect after penetration; in fact, immunoevasion/depression processes in this host–parasite system occur between 30 and 120 min post-infection [25,29].

### 2.4. Western Immunoblotting of Fat Body Protein Extracts

Larvae were sterilized in 70% ethanol and anesthetized before fat body extraction. Three larvae for each experimental group were pooled. Fat bodies were isolated using ice-cold sterile phosphate buffered saline PBS solution with the addition of drops of N-phenylthiourea (PTU). Isolated fat bodies were transferred into tubes containing PBS/PTU solution and washed with HEPES buffer (50 mM HEPES, 1 mM PMSF, 10 mM EDTA, 1X protease inhibitor cocktail, pH 7.4) before storage at −20 °C. To obtain protein extracts, fat bodies were thawed on ice and homogenized using a tissue homogenizer. Samples were then centrifuged at 12,000× *g* for 20 min at 4 degrees, and the protein concentration was estimated using the Bradford method.

Before western immunoblotting, samples containing 100 μg of total protein were diluted in Laemmli buffer (300 mM Tris-HCl, 10% glycerol, 2% SDS, 0.04% β-mercaptoethanol, pH 6.8) and the proteins were denatured for 5 min at 95 °C. The samples were then separated on 10% SDS-PAGE gels, electroblotted onto the PVDF membrane, blocked with 5% bovine serum albumin in TBS-TWEEN, probed with a rabbit anti-HSP90 antibody, diluted 1:1000 in BSA-TBS-TWEEN as the primary antibody, followed by a goat anti-rabbit HRP-conjugated secondary antibody, diluted 1:2500 in BSA-TBS-TWEEN. Subsequently, membranes were stripped using Restore Western Blot Stripping Buffer, according to the manufacturer’s specifications (Thermo Fisher, Waltham, MA, USA), and probed with a primary anti-β-actin diluted 1:1000, followed by a goat anti-rabbit HRP-conjugated secondary antibody diluted 1:2500 in BSA-TBS-TWEEN (used as a loading control). Immune complexes were visualized using the SuperSignal West Pico PLUS chemiluminescent substrate according to the manufacturer’s specifications (Bio-Rad, Hercules, CA, USA). Image acquisition was performed using Uvitec Alliance Q9 and band densitometric analyses were performed using Nine Alliance Q9 Uvitec software v18.16c.x64.

### 2.5. Statistical Analysis

Data are presented as mean ± standard deviation (SD). The normality of the data distribution was confirmed with the Shapiro–Wilk test. Statistical significance was determined using a one-way analysis of variance (ANOVA) followed by Dunnett’s post hoc test. A *p*-value of <0.05 was considered statistically significant. Adjusted *p*-values are reported in Table S1 Supplementary Materials (SM). All statistical analyses were conducted using GraphPad Prism software (version 9.0; GraphPad Software Inc., La Jolla, CA, USA).

## 3. Results

**Figure 1:**
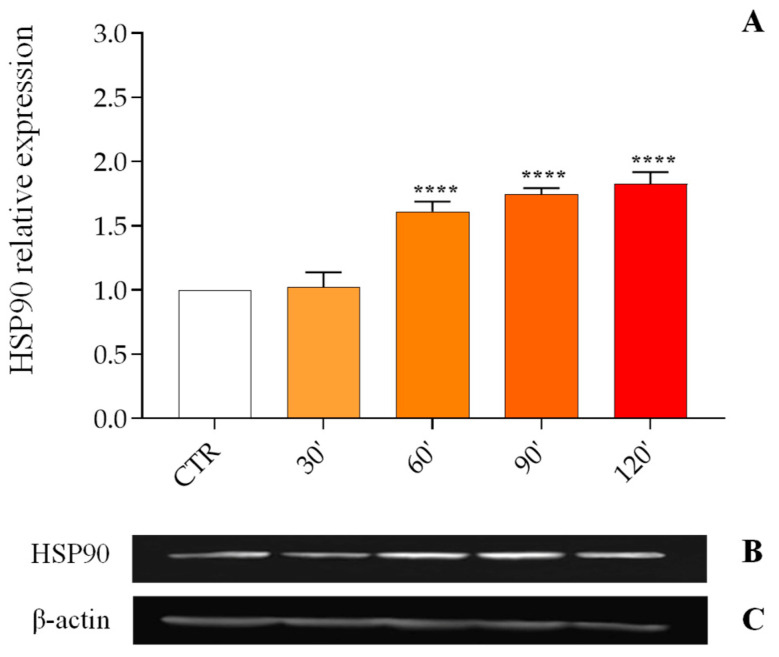
Relative expression of the HSP90 protein in the fat body following thermal stress at different time points (**Panel A**). Data are expressed as mean ± SD (n = 3) of the optical density of the HSP90 protein band (**Panel B**) versus the housekeeping protein β-actin (**Panel C**) under different experimental conditions, normalized to values obtained in the control group. **** *p* < 0.0001 vs. CTR by one-way ANOVA with Dunnett’s post hoc test.

**Figure 2:**
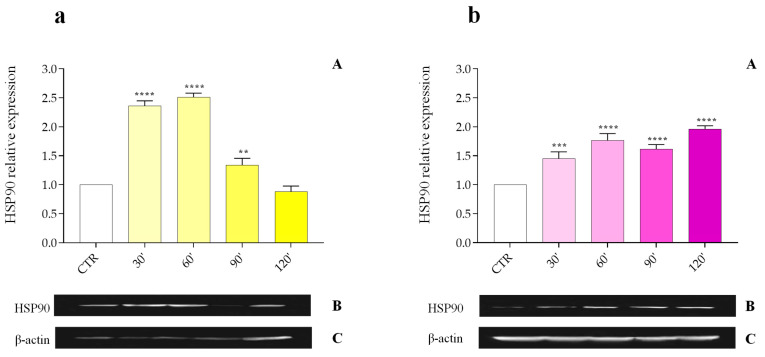
(**a**,**b**). Relative expression of the HSP90 protein in the fat body following infection with the non-entomopathogenic Gram-positive microorganism *M. luteus* (**a**, **Panel A**) and the non-entomopathogenic Gram-negative microorganism *E. coli* at different time points post-infection (**b**, **Panel A**). Data are expressed as mean ± SD (n = 3) of the optical density of the HSP90 protein band (**a**,**b**, **Panel B**) versus the housekeeping protein β-actin under different experimental conditions, normalized to values obtained in the control group (**a**,**b**, **Panel C**). **** *p* < 0.0001 vs. CTR; *** *p* < 0.001 vs. CTR; ** *p* < 0.01 vs. CTR by one-way ANOVA with Dunnett’s post hoc test.

**Figure 3:**
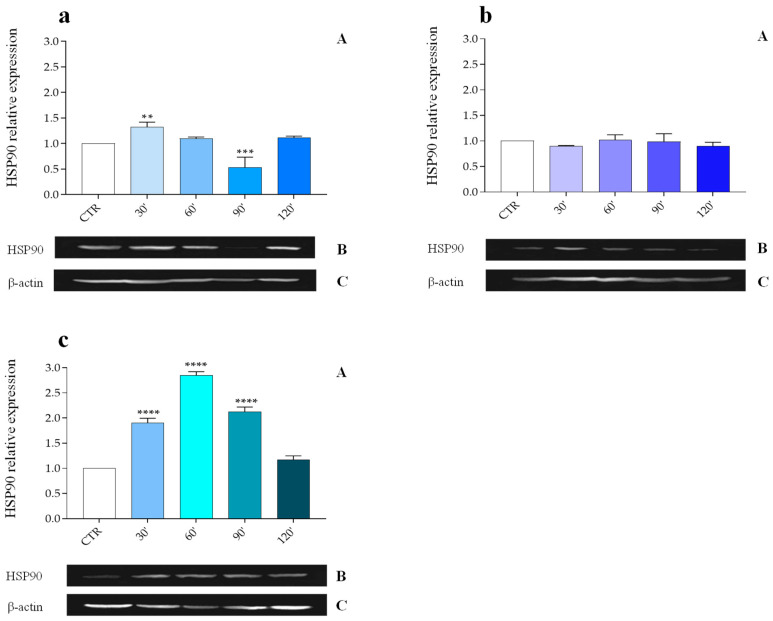
(**a**–**c**). Relative expression of the HSP90 protein in the fat body following injection with the entomopathogenic nematode *S. carpocapsae* (**a**, **Panel A**), with cold-killed *S. carpocapsae* (**b**, **Panel A**), and with the surface-treated *S. carpocapsae* (**c**, **Panel A**), at various time points post-infection. Data are expressed as mean ± SD (n = 3) of the optical density of the HSP90 band (**a**–**c**, **Panel B**) versus the housekeeping protein β-actin (**a**–**c**, **Panel C**) under different experimental conditions, normalized to values obtained from the control group. **** *p* < 0.0001; *** *p* < 0.001 vs. CTR; ** *p* < 0.01 vs. CTR by one-way ANOVA with Dunnett’s post hoc test.

**Figure 4:**
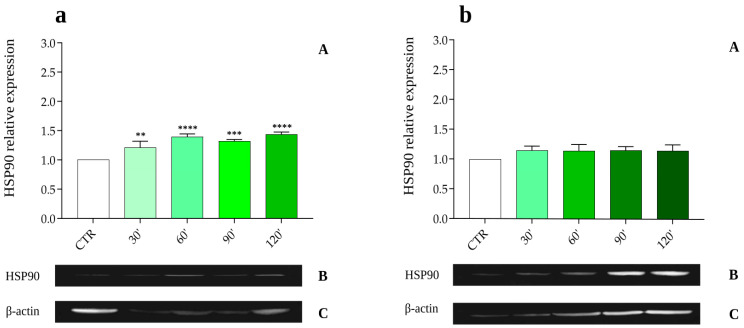
(**a**,**b**). Relative expression of the HSP90 protein in the fat body following infection with the entomopathogenic Gram-negative bacterium *X. nematophila* (**a**, **Panel A**) and with the surface-treated entomopathogenic Gram-negative bacterium *X. nematophila* (**b**, **Panel A**) at various time points post-infection. Data are expressed as mean ± SD (n = 3) of the optical density of the HSP90 band (**a**,**b**, **Panel B**) versus the housekeeping protein β-actin (**a**,**b**, **Panel C**) under different experimental conditions, normalized to values obtained from the control group. **** *p* < 0.0001; *** *p* < 0.001; ** *p* < 0.01 by one-way ANOVA with Dunnett’s post hoc test.

The HSP90 protein level in the fat body of *G. mellonella* subjected to abiotic (40 °C) or biotic stress (infections with *M. luteus*, *E. coli*, *X. nematophila,* and the nematode *S. carpocapsae*) was assessed by Western blot using anti-HSP90 antibodies (Figure 1, Figure 2, Figure 3 and Figure 4, Panels B); the obtained signal was normalized against the levels of the housekeeping protein β-actin (Figure 1, Figure 2, Figure 3 and Figure 4, Panels C). 

The expression levels of HSP90 were analyzed after stress at 40 °C (Figure 1, Panel A); thermal treatment resulted in an increase in the protein relative expression (PRE) of the target protein from 1.61 ± 0.079 at 60 min to 1.83 ± 0.093 at 120 min.

Regarding biotic stresses, HSP90 is affected by infections with non-entomopathogenic bacteria such as *M. luteus* and *E. coli*.

*M. luteus* (Figure 2a, Panel A) induces a marked increase in the relative expression of protein at 30 min (2.36 ± 0.085 PRE) and 60 min (2.51 ± 0.071 PRE) after infection. The expression level decreases after 90 min (1.34 ± 0.115 PRE) until 120 min (0.88 ± 0.095 PRE) when it is comparable with the control group.

*E. coli* infection results in a general increase in HSP90 levels (Figure 2b, Panel A), rising from 1.45 ± 0.118 PRE at 30 min to 1.96 ± 0.055 PRE at 120 min post-infection. In this case, the quantitative level of HSP90 is more uniform compared to that observed with the Gram-positive bacterium *M. luteus* (Figure 2a, Panel A).

In contrast to data obtained from previous bacterial infections, the presence of the nematocomplex *S. carpocapsae* (Figure 3a, Panel A) does not appear to significantly affect the expression of HSP90 in the fat body of *G. mellonella*, when compared with the control. At early post-infection time points (30 min), a slight increase in the HSP90 protein expression is observed (1.33 ± 0.093 PRE), followed by a substantial decrease at 90 min (0.54 ± 0.198 PRE) and a partial recovery at 120 min (1.12 ± 0.031 PRE) post-infection.

Additional tests were conducted using non-viable (cold-killed) *S. carpocapsae* nematodes to rule out any effects from excretion/secretion products or symbiotic bacteria in the absence of HSP90 activation. Injection of cold-killed nematodes showed no overall increase in HSP90 expression (Figure 3b, Panel A) across the time points analyzed. 

After confirming that non-viable nematodes did not alter HSP90 expression levels, the potential role of the parasite body surface (epicuticle) was assessed. The nematode body surface was treated to remove lipids from the epicuticle, known to play a key role in immunoevasion strategies. Assays with surface-treated nematodes demonstrated a marked increase in HSP90 expression, rising significantly from 30 min (1.9 ± 0.095 PRE) to 60 min (2.85 ± 0.070 PRE) post-injection. Following this peak, HSP90 levels gradually declined, reaching 2.13 ± 0.093 PRE at 90 min, and returning to baseline by 120 min (1.17 ± 0.076 PRE), similar to control levels (Figure 3c, Panel A).

The effects of the symbiotic and entomopathogenic bacterium *X. nematophila* (previously isolated from *S. carpocapsae*) on HSP90 expression were also investigated (Figure 4). The results show a moderate but significant increase in HSP90 levels, particularly at 60, 90, and 120 min post-infection (Figure 4a, Panel A), with relative expression levels of 1.39 ± 0.051, 1.32 ± 0.025, and 1.43 ± 0.040 PRE, respectively.

In addition, to determine whether the surface components *X. nematophila* may be responsible for the lack of increase in HSP expression levels, symbiont bacteria were treated with a high ionic strength buffer to remove membrane proteins weakly associated with the cell surface and were then injected into *G. mellonella* larvae. Analysis of the expression levels of HSPs over the time interval considered (30–120 min) showed no significant differences between the treated bacteria and control groups (Figure 4b, Panel A).

**Figure 5:**
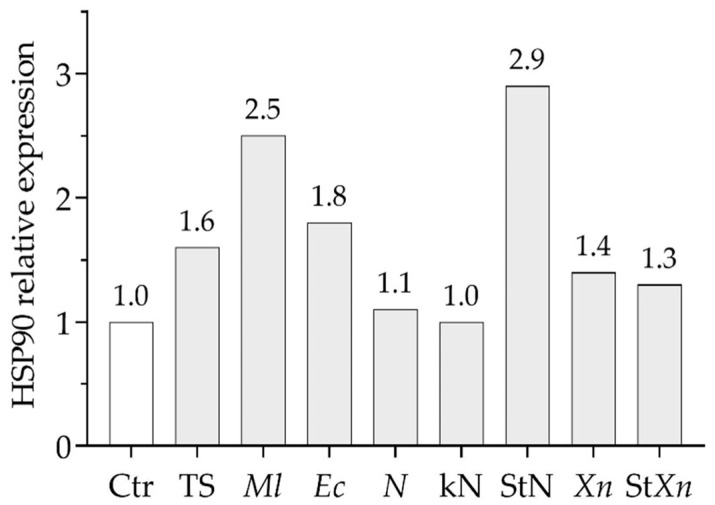
Comparison of HSP90 relative expression in the fat body at 60 min after treatments. Ctr: control; TS: thermal shock; *Ml*: *M. luteus*; *Ec*: *E. coli*; N: *S. carpocapsae*; KN: cold-killed *S. carpocapsae*; StN: surface-treated *S. carpocapsae*; *Xn*: *X. nematophila*; St*Xn*: surface-treated *X. nematophila*.

Figure 5 shows a comparison of HSP90 expression levels 60 min after the different abiotic and biotic treatments.

**Figure 6:**
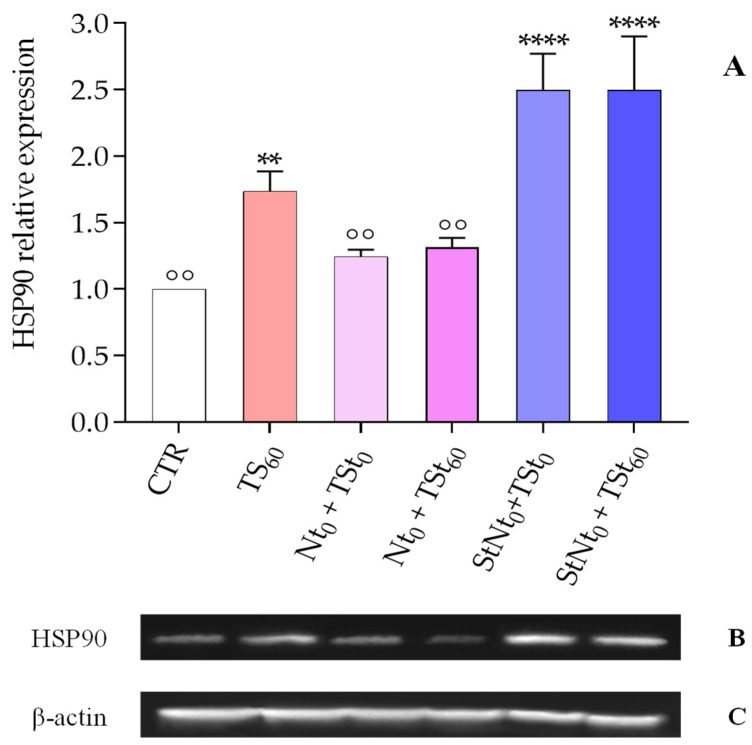
Combined effects of thermal shock and *S. carpocapsae* infection (**Panel A**). Relative expression of the HSP90 protein in the fat body after infection with live (N) or surface-treated (StN) nematodes, both subjected to thermal stress at various times. Data are expressed as the mean ± SD (n = 3) of the optical density of the bands of the protein of interest HSP90 (**Panel B**) versus the reference β-actin (**Panel C**) under the different experimental conditions, normalized to the values obtained in the control group. TS: thermal stress 40 °C; N: live nematode; StN: surface-treated nematode. **** *p* < 0.0001 vs. CTR; ** *p* < 0.01 vs. CTR; °° *p* < 0.01 vs. TS_60_ by one-way ANOVA with Dunnett’s post hoc test.

The effects on the expression levels of HSP90 were also evaluated by combining abiotic and biotic stresses at various times (Figure 6). Larvae were injected with viable or surface-treated nematodes and heat stress was induced at t = 0 (immediately after nematode injection) or delayed (60 min after infection).

The results indicated that heat shock (40 °C) administered either simultaneously (t_0_) or after 60 min (t_60_) with living nematodes results in a marked reduction of the expression of the target protein (Figure 6, Nt_0_ + TSt_0_ and Nt_0_ + TSt_60_) when compared with heat shock alone (Figure 6, TS_60_). When the combined tests were performed by injecting surface-treated nematodes (Figure 6, StNt_0_ + TSt_0_ and StNt_0_ + TSt_60_), the protein was overexpressed at a level comparable to that observed after injection of surface-treated nematodes at standard conditions (26 °C), after 60 min (Figure 3, Panel C), with HSP90 significantly increased compared with heat treatment alone, independently of the time of temperature stress application (Figure 6, Panel A, TS_60_).

## 4. Discussion

The study of biopesticide efficacy requires an in-depth understanding of the molecular mechanisms regulating the immune pathways of harmful insects. The use of biological control, particularly the application of entomopathogenic nematodes such as *S. carpocapsae*, is a highly effective and environmentally sustainable approach to pest management [30]. These organisms can control pest diffusion naturally, offering an effective, environmentally friendly approach to pest control. Unlike synthetic compounds, biological agents do not persist in the environment, thereby preserving biodiversity and decreasing the likelihood of resistance development among pest populations; moreover, the adaptability of entomopathogenic nematodes to different environmental conditions, including those related to climate change, underscores their potential as bioinsecticides, even in the context of global warming.

Variations in environmental temperature can affect the effectiveness of biological control measures, as reported by Mastore et al. on *Drosophila suzukii*, which showed that daily temperature fluctuations alter the efficacy of bioinsecticides such as *Bacillus thuringiensis*, *S. carpocapsae*, *Steinernema feltiae*, and *H. bacteriophora* [31]. The choice of biological insecticides that exhibit beneficial properties when used to manage pest populations in specific locations is a central topic of investigation to date.

The expression of heat shock proteins is widely recognized as being influenced not only by temperature variations but also by biotic stress. In this study, a targeted analysis of heat shock protein 90 (HSP90) was performed in order to assess its expression in response to immunoevasive entomopathogen nematode *S. carpocapsae* and its symbiotic bacterium *X. nematophila*. Given the role of temperature in modulating the efficacy of entomopathogenic nematodes, the potential correlation between their presence, thermal fluctuations, and HSP expression levels was investigated.

Initially, HSP levels were evaluated both following thermal shock and after infection with non-entomopathogenic Gram-positive (*M. luteus*) and Gram-negative (*E. coli*) bacteria.

As expected, both temperature and non-entomopathogenic microorganisms influence the expression of HSP90, leading to a significant increase in its physiological levels. The effects of thermal stress and non-entomopathogenic bacteria are also confirmed by the study of Wojda and colleagues, where levels of the HSP90 protein and an unknown 55-kDa protein were increased in *G. mellonella* larvae subjected to heat shock or infection, and pretreatment of larvae with the HSP90 inhibitor 17-DMAG resulted in even higher expression of AMP genes and improved survival after *Pseudomonas aeruginosa* infection. Moreover, larvae exposed to heat shock (38 °C) showed a stronger immune response to microbial infection, with increased expression of antimicrobial peptide genes and, consequently, increased antimicrobial activity in the hemolymph, compared with larvae maintained at 28 °C [9].

To assess HSP90 levels following entomopathogenic nematode infection, living nematodes, cold-killed nematodes (subjected to a freezing cycle), or nematodes treated with agents to remove epicuticular lipids (surface-treated) were injected into *G. mellonella* larvae.

We found that injection of live or cold-killed nematodes resulted in minimal or no effect on HSP90 expression, respectively. Conversely, the injection of nematodes whose lipid cuticles had been irreversibly damaged by methanol/chloroform treatment led to a significant increase in HSP90 protein expression, even greater than the response observed after infection with various bacterial species. 

As shown in a study on *S. feltiae* [25,29], the cuticular lipids of this parasite play a key role in the evasion and depression of the immune system of *G. mellonella*; cuticular lipids bind specific molecules of the host hemolymph, forming a self-protein coat around the nematode, thus preventing the immune system from recognizing the parasite as foreign. This molecular disguise process prevents hemocytes from detecting and attacking the nematode, thus avoiding encapsulation and immune responses such as melanization. The observed differential expression of HSP90 can be attributed to the host immune system’s inability to effectively recognize the parasite, consequently, there is little or no overexpression of HSP90 as nematodes disguise themselves and suppress the immune system. In contrast, the nematodes with damaged lipidic epicuticles expose underlying proteins that are more readily identified as non-self by the host immune system. This different molecular body surface pattern triggers a strong immune response, leading to significant expression of HSP90; thus, it appears that the key trigger for HSP90 expression is the exposure to nematode factors, which the immune system recognizes as non-self.

Further tests were carried out to evaluate whether the effects of temperature variation could be influenced by the presence of *S. carpocapsae*. Larvae were injected with viable or surface-treated nematodes and subjected to thermal stress at 40 °C, initiated at either t_0_ or t_60_. The findings revealed that the presence of viable nematodes in the host insect’s hemolymph suppressed HSP90 overexpression, even under thermal stress, indicating potential interferences by the parasite with the host’s temperature-induced activation mechanisms. In contrast, the administration of surface-treated nematodes resulted in a significant upregulation of HSP90 compared to heat treatment alone, regardless of the timing of the thermal stress induction. The assays demonstrated that thermal stress applied to surface-treated nematode-infected larvae elicited a stronger response compared to heat shock alone, highlighting the immunoevasive or immunosuppressive properties of the nematodes that can significantly modulate HSP90 expression, even under conditions that typically promote its overexpression.

*Steinernema* spp. exists in a symbiotic relationship with the bacterium *X. nematophila* [32], serving as a vector for the bacteria while suppressing host immunity to facilitate the lethal effects of the symbiont. This process aids the symbiotic bacteria in inducing sepsis, leading to host death. Thus, we administered *X. nematophila*, isolated from *S. carpocapsae* to *G. mellonella* larvae to evaluate HSP90 levels. Following infection with *X. nematophila*, HSP90 expression was significantly reduced, particularly when compared to the stimulation induced by non-entomopathogenic bacteria such as *E. coli* and *M. luteus*. This reduced HSP90 expression, comparable to physiological baseline levels, and like results with nematodes, may be attributed to the immunoevasive and immunosuppressive properties of *X. nematophila* [32].

Normally, bacterial cells present in the hemolymph of insects are phagocytosed or nodulated by circulating hemocytes; in the case of *X. nematophila*, the phagocytic efficiency is extremely low, as observed in *Locusta migratoria,* in which hemocytes removed the bacteria with an efficiency of 10% [33]. Previous research on *G. mellonella* demonstrated that the immunoevasive abilities of *X. nematophila* were altered following high ionic strength treatment of the bacteria. The removal of weakly bound surface proteins, referred to as *Xenorhabdus* outer proteins (XoPs), resulted in the loss of their elusive properties, leading to a significant increase in phagocytosis [34].

In this work, the removal of *Xenorhabdus* external proteins, via treatment with a high ionic strength buffer, did not induce significant changes in HSP90 expression. Although these surface factors appear to be responsible for the evasive strategies of the bacterium, which elude immunological recognition, they do not appear to be directly involved in the HSP activation pathways.

Consistent with our findings, Li et al. reported a reduction in HSP90 gene expression in the midgut of the beetle *Holotrichia parallela* when exposed to the nematode *Heterorhabditis beicherriana* [35]. Similarly, Ding et al. observed the downregulation of HSP90 genes in the lepidopteran *Glyphodes pyloalis* during the early stages of parasitization by the parasitoid wasp *Aulacocentrum confusum* [36].

Overexpression of HSP90 following entomopathogenic bacterial infections has been reported in the literature; for instance, Wojda and colleagues demonstrated that infection with *B. thuringiensis* (5 × 10^3^ cells) leads to a marked overexpression of HSP90; this upregulation suggests that HSP90 might be a critical component of the stress response triggered by these bacteria in *G. mellonella*. Interestingly, when the HSP90 inhibitor 17-DMAG was introduced, an improved defensive response was observed. This enhanced immune efficacy against the entomopathogenic bacterium appears to be related to increased lysozyme activity and increased expression of antimicrobial peptides (AMPs). The results suggest that while HSP90 is upregulated during infection, its inhibition may paradoxically lead to a more robust immune response by modulating other pathways that enhance larval defense mechanisms [37]. 

In contrast, Richards et al. reported an upregulation of heat shock proteins (HSPs) with enhanced humoral and cellular immune responses in various tissues of the lepidopteran *Mamestra brassicae* following infection with entomopathogenic fungi [38].

The expression of HSP90 has also been investigated after fungi exposure; Wrońska and colleagues infected *G. mellonella* larvae with *Conidiobolus coronatus* to monitor HSP90 levels; the results show no significant changes in HSP90 expression in the first 24 h, indicating that the early stages of fungal infection do not immediately trigger a stress response involving HSP90. However, a significant increase in HSP90 expression was observed 48 h after infection, suggesting a delay in the involvement of HSP90 in the immune response to fungal pathogens [7]. This temporal shift in HSP90 expression underscores the complexity of the immune response in *G. mellonella* and highlights how the nature and timing of pathogen exposure can differentially regulate stress proteins.

However, different observations on the function of HSPs in relation to immunity have been reported in the literature, but we have to consider the fact that most of the studies are carried out on different host–entomopathogen systems, which implement their own evasive or suppressive strategies.

This evidence, along with the data produced in the present study, suggests that HSP90 may serve as a key modulator in the response to biopesticides and non-self-organisms; HSP90 is overexpressed in response to non-entomopathogenic bacteria or nematodes, but only when the latter, after the alteration of the body surface, are recognized as non-self by the insect’s immune system. Indeed, when the cuticle is compromised, other cuticular surface proteins (i.e., cuticlins) or different lipids are exposed, allowing the immune system to recognize the invader and trigger the upregulation of HSP90.

## 5. Conclusions

The potential interaction between biotic stresses and environmental variability is an important aspect to consider in host–parasite dynamics. This study could provide a starting point for investigating these complex interactions in different systems, including a range of insect species and entomopathogenic organisms or microorganisms.

Specifically, this study examines the interactions between biological control agents (*S. carpocapsae*) and the immune responses of host insects (*G. mellonella*), with emphasis on the expression of HSP90. The findings indicate that HSP90 expression in *G. mellonella* is influenced by the type of pathogen or parasite involved, suggesting a potential relationship between HSP90 expression levels and the host’s immune response following infection. The effectiveness of insect control is determined by both the mechanisms employed by bioinsecticides and the resistance of the host insect’s immune defenses.

Climate change, particularly fluctuations in environmental temperature, could affect host–parasite interactions in biological control by altering HSP expression. Such changes may subsequently modify the immune response of insects exposed to bioinsecticides, including entomopathogenic nematodes. Further investigation is required to better understand the correlation between HSP activation pathways, host immune mechanisms, and the immunoevasion or immunosuppression strategies employed by different entomopathogen species.

## Data Availability

The data presented in this study are available upon request to the corresponding author.

## Supplementary Materials

The following supporting information can be downloaded at https://www.mdpi.com/article/10.3390/insects16020201/s1, Table S1: Adjusted *p*-values and significance of the different experimental groups against the reference group.
