# Supplementary material for: The Expression Levels of Heat Shock Protein 90 (HSP90) in Galleria mellonella Following Infection with the Entomopathogenic Nematode Steinernema carpocapsae and Its Symbiotic Bacteria Xenorhabdus nematophila
Source: Insects. 2025 Feb 12;16(2):201. doi: 10.3390/insects16020201 (PMC11856864; doi:10.3390/insects16020201)
Supplement: Supplementary file 1 [file insects-16-00201-s001.zip › insects-3410219-supplementary.pdf]

Banfi et al. The Expression Levels of Heat Shock Protein 90 (HSP90) in Galleria mellonella Following Infection with the Entomopathogenic Nematode Steinernema carpocapsae and Its Symbiotic Bacteria Xenorhabdus nematophila

SUPPLEMENTARY MATERIALS

**Dunnett's multiple comparisons test**	**Summary**	**Adjusted P Value**
**Thermal stress**		
CTR vs. 30'	ns	0,9932
CTR vs. 60'	****	<0,0001
CTR vs. 90'	****	<0,0001
CTR vs. 120'	****	<0,0001
Micrococcus luteus		
CTR vs. 30'	****	<0,0001
CTR vs. 60'	****	<0,0001
CTR vs. 90'	**	0,0018
CTR vs. 120'	ns	0,3105
Escherichia coli		
CTR vs. 30'	***	0,0002
CTR vs. 60'	****	<0,0001
CTR vs. 90'	****	<0,0001
CTR vs. 120'	****	<0,0001
Steinernema carpocapsae		
CTR vs. 30'	**	0,0081
CTR vs. 60'	ns	0,5718
CTR vs. 90'	***	0,0007
CTR vs. 120'	ns	0,4496
**Cold-killed *S. carpocapsae***		
CTR vs. 30'	ns	0,4976
CTR vs. 60'	ns	0,9978
CTR vs. 90'	ns	0,9990
CTR vs. 120'	ns	0,4714

**Surface-treated *S. carpocapsae***		
CTR vs. 30'	****	<0,0001
CTR vs. 60'	****	<0,0001
CTR vs. 90'	****	<0,0001
CTR vs. 120'	ns	0,0567
Xenorhabdus nematophila		
CTR vs. 30'	**	0,0041
CTR vs. 60'	****	<0,0001
CTR vs. 90'	***	0,0002
CTR vs. 120'	****	<0,0001
**Surface-treated *Xenorhabdus nematophila***		
CTR vs. 30'	ns	0,1940
CTR vs. 60'	ns	0,0993
CTR vs. 90'	ns	0,1395
CTR vs. 120'	ns	0,1082
**Combined effects of thermal shock and *S. carpocapsae* infection**		
CTR vs. TS_{60}	**	0,0046
CTR vs. $StNt_0$+TSt_0	****	<0,0001
CTR vs. $StNt_0$ + TSt_{60}	****	<0,0001
CTR vs. Nt_0 + TSt_0	ns	0,5031
CTR vs. Nt_0 + TSt_{60}	ns	0,2917
TS_{60} vs. CTR	°°	0,0046
TS_{60} vs. $StNt_0$+TSt_0	°°	0,0036
TS_{60} vs. $StNt_0$ + TSt_{60}	°°	0,0036
TS_{60} vs. Nt_0 + TSt_0	ns	0,0565
TS_{60} vs. Nt_0 + TSt_{60}	ns	0,1132

Table S1 Supplementary Materials. Adjusted P-values and significance of the different experimental groups against the reference group.
